# Interferon-Stimulated Genes—Mediators of the Innate Immune Response during Canine Distemper Virus Infection

**DOI:** 10.3390/ijms20071620

**Published:** 2019-04-01

**Authors:** Daniela Klotz, Ingo Gerhauser

**Affiliations:** Department of Pathology, University of Veterinary Medicine Hannover, 30559 Hannover, Germany; Daniela.Klotz@tiho-hannover.de

**Keywords:** canine distemper virus, demyelination, immunofluorescence, immunohistochemistry, interferon-regulated factor, interferon-stimulated gene 15, microarray analysis, 2′-5′-oligoadenylate synthetase, protein kinase R, type I interferon

## Abstract

The demyelinating canine distemper virus (CDV)-leukoencephalitis represents a translational animal model for multiple sclerosis. The present study investigated the expression of type I interferon (IFN-I) pathway members in CDV-induced cerebellar lesions to gain an insight into their role in lesion development. Gene expression of 110 manually selected genes in acute, subacute and chronic lesions was analyzed using pre-existing microarray data. Interferon regulatory factor (IRF) 3, IRF7, signal transducer and activator of transcription (STAT) 1, STAT2, MX protein, protein kinase R (PKR), 2′-5′-oligoadenylate synthetase (OAS) 1 and interferon-stimulated gene (ISG) 15 expression were also evaluated using immunohistochemistry. Cellular origin of STAT1, STAT2, MX and PKR were determined using immunofluorescence. CDV infection caused an increased expression of the antiviral effector proteins MX, PKR, OAS1 and ISG15, which probably contributed to a restricted viral replication, particularly in neurons and oligodendrocytes. This increase might be partly mediated by IRF-dependent pathways due to the lack of changes in IFN-I levels and absence of STAT2 in astrocytes. Nevertheless, activated microglia/macrophages showed a strong expression of STAT1, STAT2 and MX proteins in later stages of the disease, indicating a strong activation of the IFN-I signaling cascade, which might be involved in the aggravation of bystander demyelination.

## 1. Introduction

Canine distemper virus (CDV) is an enveloped, single-stranded, negative-sense RNA virus [1,2], which can infect terrestrial and aquatic carnivores via the oropharyngeal route [3,4]. The clinical course of the disease depends on the CDV strain as well as the immune status and age of the infected host [2,4]. CDV-infected animals can either trigger a strong humoral and cellular immune response between the 8th and 14th day after infection and recover or, in the case of a weak immune response, die or develop a persistent infection [4,5]. Clinically, canine distemper is predominantly characterized by catarrhal respiratory and enteric disease signs, but a systemic form affecting also the central nervous system (CNS) and unusual manifestations including the so-called “hard pad” disease can occur [4]. The clinical disease of canine distemper is similar in pathogenesis and symptoms to measles virus infection in humans [4]. Neurologic manifestations of canine distemper can be caused by gray and/or white matter disease [4,5,6,7]. The rarer polioencephalitis is divided into “old dog encephalitis”, “post-vaccination distemper encephalitis” and “inclusion body polioencephalitis” [6,7]. However, the nervous form usually manifests as leukoencephalitis within the cerebellum [5]. The lesions can be divided into acute (vacuolation of the white matter and mild astrogliosis), subacute (vacuolation and demyelination with mild or no inflammation) and chronic changes (demyelination with moderate to severe inflammation) [8]. The demyelinating distemper leukoencephalitis also represents a naturally occurring animal model for human multiple sclerosis (MS) due to similar histopathological findings and pathogenic mechanisms [3,9,10].

Interferons (IFNs) are signaling proteins, which can be classified into three major types based on their specific receptor binding: type I (IFNα and IFNβ), type II (IFNγ) and type III (IFNλ) [11,12]. Type I IFNs (IFN-I) play an important role in the innate immune response and have antiviral, antiproliferative and immunomodulatory capacities, which are already used in the therapy of human and animal disorders [13,14]. IFNγ is primarily produced in later stages of the immune response by B cells, cytotoxic T cells and T helper (Th) cells and represents the main activator of macrophages [15]. IFNλ also has antiviral and antiproliferative effects but its role during canine distemper and therapeutic potential seems to be limited [14,16,17,18,19,20]. While in human medicine administering IFN-I in MS is part of the standard therapy [21], the therapeutic effect of IFN-I on the outcome of a CDV infection in dogs is still widely undetermined [14,22].

The IFN-I pathway is initiated by the detection of typical structural elements of infectious agents (pathogen-associated molecular patterns; PAMPs), which can be recognized by so-called pattern recognition receptors (PRRs) [23]. Single- and double-stranded RNA produced during viral replication can be detected by toll-like receptors (TLRs), protein kinase R (PKR) and the family of the retinoic acid-inducible gene-I-like receptors (RLRs) including retinoic acid-inducible gene 1 (RIG1) and melanoma differentiation-associated gene 5 (MDA5), which are bound to endosomal membranes or localized in the cytoplasm [24,25,26]. After binding to viral RNA, these receptors recruit specific adapter proteins like Toll/IL-1 receptor domain containing adapter-inducing IFNβ (TRIF), myeloid differentiation primary response gene 88 (MyD88) and IFNβ promoter stimulator (IPS-1) as well as second messengers to activate IFN regulatory factor (IRF) 3 and IRF7, which induce the transcription of IFN-I [23,27,28,29,30]. IFN-I bind in an autocrine or paracrine fashion to their specific receptor, consisting of the subunits IFNAR1 and IFNAR2, to trigger signaling pathways that inhibit viral replication [31]. The receptor-associated kinases Janus kinase 1 (JAK1) and tyrosine kinase 2 (TYK2) phosphorylate the transcription factors, signal transducer and activator of transcription (STAT) 1 and STAT2, which then associate with IRF9 to form a heterotrimeric complex termed interferon-stimulated gene factor 3 (ISGF3) [32,33,34,35]. After translocation into the nucleus, ISGF3 induces the transcription of numerous IFN-stimulated genes (ISGs), which have an IFN-stimulated response element (ISRE) in their promoter site [30,35,36,37]. ISGs including MX proteins, 2′-5′-oligoadenylate synthetase (OAS), PKR and ISG15 play an important role in the resistance to viral infections due to their inhibitory effect on viral transcription, translation and release [30,38,39]. In addition, non-canonical aspects of JAK-STAT signaling are described, in which STAT2 homodimers mediate mechanisms of defense against pathogens that impede STAT1 signaling [40,41].

The presence of IFN-I in the cerebrospinal fluid (CSF) of CDV-infected dogs has been known for a long time and was suggested to be used as a marker of virus persistence, but IFN-I CSF levels are generally low and not always present [42,43]. An immunohistochemical study demonstrated strong expression of MX proteins in CDV-induced brain lesions [44]. Moreover, recent studies have shown that the virulence of CDV depends on the suppression of the IFN-I signaling pathway via interference with MDA5 and STAT2 signaling [45]. Interestingly, dogs exhibit a relatively high OAS serum activity compared to other animal species such as cats, rabbits and guinea pigs [46]. However, ISG expression has hardly been investigated in the CNS of CDV-infected dogs so far. The present study characterized the expression of several IFN-I pathway members on the gene and protein level in order to contribute to the understanding of the role of the innate immune response in the initiation and progression of CDV-infected cerebellar lesions.

## 2. Results

### 2.1. Microarray

**Figure 1:**
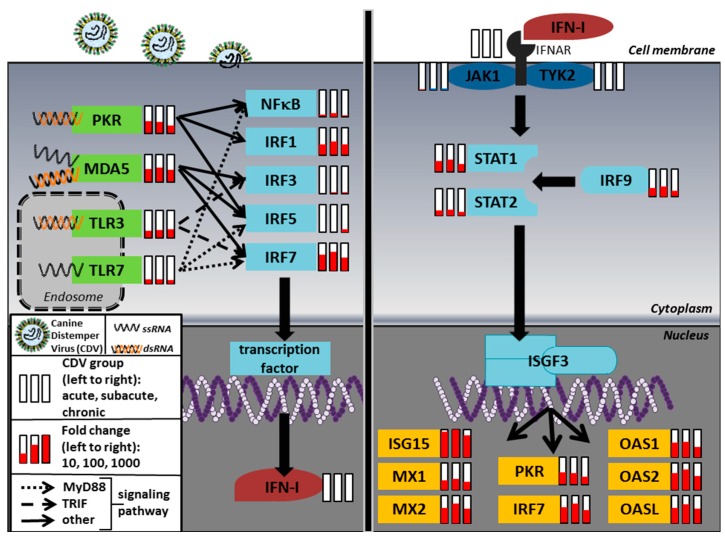
Transcriptional changes of type I interferon (IFN-I) signaling pathway members in the cerebellum of canine distemper virus (CDV)-infected compared to control dogs. Left side: RNA of CDV is recognized by pattern recognition receptors (PKR, MDA5, TLR3, TLR7), which leads to the activation of transcription factors (NFκB, IRF1, IRF3, IRF5, IRF7). These transcription factors translocate into the nucleus and induce IFN-I expression. Right side: Receptor binding of IFN-I activates the JAK-STAT signaling pathway leading to the formation of STAT1/STAT2 heterodimers in the cytoplasm. These heterodimers form a complex with IRF9, termed interferon-stimulated gene factor 3 (ISGF3). In the nucleus, ISGF3 induces the transcription of interferon-stimulated genes including ISG15, MX1, MX2, PKR, IRF7, OAS1, OAS2 and OASL. IRF7 also acts in a positive feedback loop to stimulate IFN-I expression. Columns behind the gene symbols display fold changes in three different stages of CDV-infection (acute; subacute; chronic). Fold changes are shown on a logarithmic scale.

The original microarray analysis performed by Ulrich et al. (2014) revealed 780 differentially expressed probe sets and the dominating change was an up-regulation of genes related to the innate and the humoral immune response [47]. Of 110 manually selected genes of the IFN-I pathway, 57% (63/110) were up-regulated in a relevant manner (significant fold change > 1.5) at least in one group of CDV-induced lesions, and gene expression usually peaked in the subacute stage (Figure 1, Table 1, Table S1). Toll-like receptor (Tlr) 2, Tlr3 and Tlr7 (fold change up to 4.52, 8.00 and 2.83) were moderately increased, whereas Pkr and Mda5 were highly up-regulated (fold change up to 15.62 and 33.76). Only a mild increase in Irf3, Irf5 and nuclear factor of kappa light polypeptide gene enhancer in B-cells (Nfkb) 1 gene expression (fold change < 2) was found, whereas Irf1 and Irf7 were strongly induced (fold change up to 30.13 and 113.54). No transcriptional changes were found in IFN-I gene expression and Ifnar genes were only mildly increased (fold change < 2) in the subacute stage of CDV-infection. Similarly, most signal transducers including Jak1, Socs1 and Tyk2 did not show significant transcriptional changes. Nevertheless, Stat1, Stat2 and Irf9 were moderately up-regulated (fold change up to 19.19, 4.57 and 11.44). The classical ISGs were strongly induced by CDV-infection with particularly high levels for Isg15, Ifi44l, Isg56, Oas2 and Mx2 (fold change up to 928.35, 419.93, 179.05, 173.57 and 147.59). Additionally, several MHC class I and class II genes were highly up-regulated (fold change up to 135.55 and 30.97). An overview of fold changes for selected interferon-dependent genes is given in Figure 1, Table 1 and Table S1.

### 2.2. Classification of Histological Lesions

**Figure 2:**
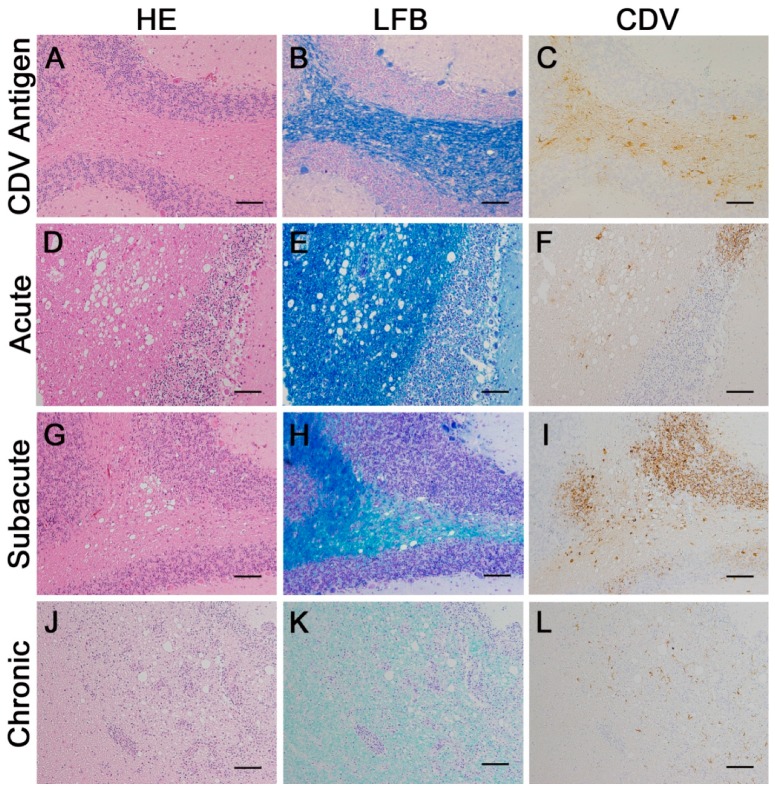
Classification of cerebellar lesions in the white matter of canine distemper virus (CDV)-infected dogs. (**A**–**C**) Group 2: antigen expression without histological lesions. (**D**–**F**) Group 3: acute lesion. (**G**–**I**) Group 4: subacute lesion. (**J**–**L**) Group 5: chronic lesion with prominent inflammation. (**A**,**D**,**G**,**J**) hematoxylin and eosin (HE)-staining; (**B**,**E**,**H**,**K**) luxol fast blue (LFB) staining; (**C**,**F**,**I**,**L**) CDV. Immunohistochemistry visualized by the avidin-biotin-peroxidase complex (ABC) method with 3,3′-diaminobenzidine as substrate and counterstaining with Mayer’s hematoxylin. Bars, 100 µm.

Lesions (130) found in cerebellar tissue of 15 CDV-infected dogs were analyzed using HE- and LFB-stainings and CDV immunohistochemistry. In 16 lesions, CDV antigen changes but not histological ones were detected (group 2). Twenty-six acute lesions were characterized by white matter vacuolation and absence of demyelination (group 3). Forty-eight subacute lesions showed demyelination in the LFB-staining but no inflammatory cell infiltration in the HE-staining (group 4). Forty chronic lesions demonstrated advanced demyelination, perivascular cuffing and lymphocytes in the parenchyma (group 5). In addition, 53 areas were randomly selected in the cerebellar white matter of six control dogs for evaluation (group 1). Representative pictures of the investigated lesions are shown in Figure 2 to illustrate classification.

### 2.3. Immunohistochemistry

**Figure 3:**
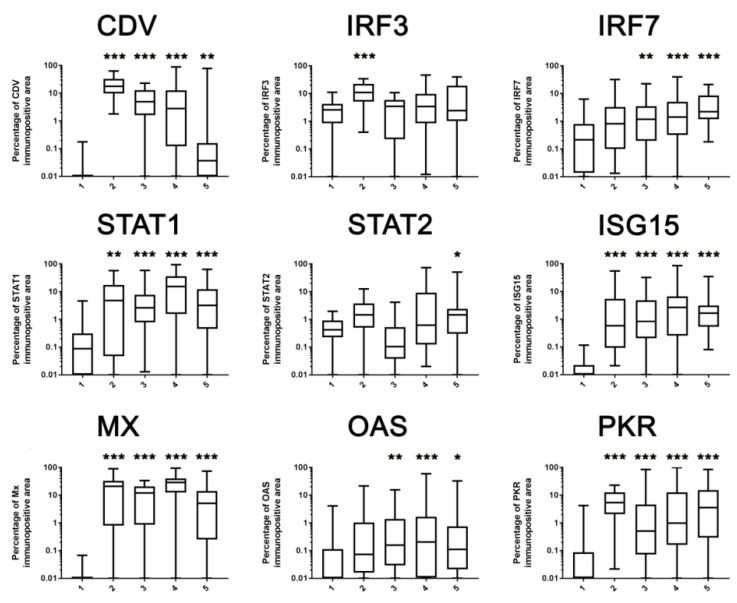
Canine distemper virus (CDV) antigen and IRF3, IRF7, STAT1, STAT2, ISG15, MX, OAS and PKR protein expression in the cerebellar white matter. Group 1 are areas in the white matter of control dogs; group 2 are foci with CDV antigen expression but without histological lesions; group 3 are acute CDV lesions; group 4 are subacute foci without inflammation; group 5 are chronic CDV lesions with inflammation. Shown are the percentages of the immunopositive area using box plots in a logarithmic scale and significant differences between the CDV-infected groups 2–5 and the control group 1 based on Kruskal-Wallis-tests and Dunn’s multiple comparison tests. * *p* < 0.05; ** *p* < 0.01; *** *p* < 0.001.

**Figure 4:**
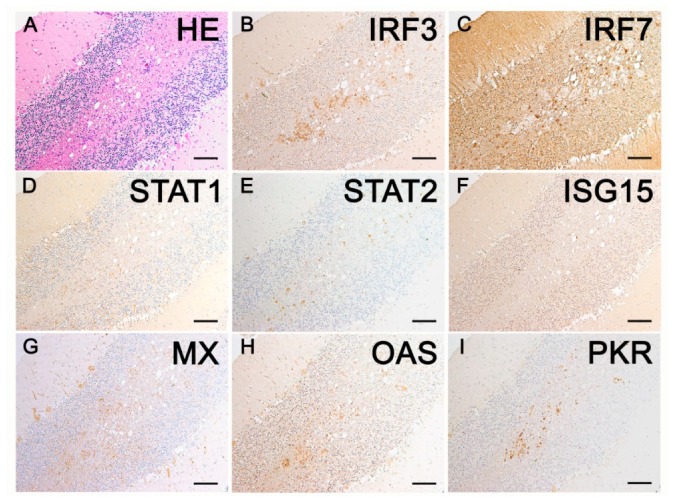
IRF3, IRF7, STAT1, STAT2, ISG15, MX, OAS and PKR protein expression in serial sections of a chronic white matter lesion in the cerebellum of a canine distemper virus-infected female mixed breed dog. (**A**) Lesion with perivascular inflammatory cells (hematoxylin and eosin (HE)-staining); (**B**) Strong IRF3 protein expression of glial cells and perivascular lymphocytes; (**C**) Strong IRF7 staining of neurons and glial cells; (**D**) STAT1 protein expression of glial cells and a few neurons; (**E**) Strong immunostaining of glial cells and Purkinje cells for STAT2 proteins; (**F**) ISG15 protein expression of granular cell layer neurons and intralesional glial cells; (**G**) Strong Mx protein expression of numerous intralesional glial and inflammatory cells as well as Purkinje cells; (**H**) Strong OAS protein expression of perivascular lymphocytes, intralesional glial cells and Purkinje cells; (**I**) Strong PKR protein expression particularly of intralesional lymphocytes. Immunohistochemistry visualized by the avidin-biotin-peroxidase complex method with 3,3′-diaminobenzidine as substrate and counterstaining with Mayer’s hematoxylin. Bars, 100 µm.

In general, immunohistochemical analysis detected an increase in the protein expression of all investigated IFN-I pathway members in CDV-induced white matter lesions (Figure 3 and Figure 4, Table 2, Figure S1).

A statistically significant up-regulation of IRF3 protein expression was only found in group 2 lesions compared to controls (*p* < 0.001). Endothelial cells, neurons and glial cells were positive for IRF3 in all investigated groups. In addition, perivascular lymphocytes present in group 5 expressed IRF3. IRF7 protein was not expressed in perivascular lymphocytes but in glial cells and neurons in infected and control groups. There was a prominent staining in the granular cell layer and a slight staining of Purkinje cells. The IRF7 protein expression was significantly up-regulated in acute (*p* = 0.006), subacute (*p* < 0.001) and chronic lesions (*p* < 0.001) and continuously increased during lesion progression. STAT1 protein expression was increased at all stages of CDV-induced leukoencephalitis (group 2: *p* = 0.003; Groups 3–5: *p* < 0.001). In CDV-infected cerebellar tissue, STAT1 proteins were mainly detected in glial cells, whereas only a few neurons and endothelial cells expressed STAT1 and perivascular cells were mainly negative. In control tissue, only single glial cells, as well as some neurons and endothelial cells, slightly expressed STAT1. STAT2 protein expression was significantly elevated only in chronic lesions (group 5: *p* = 0.02). Perivascular lymphocytes were negative for STAT2, whereas neurons, especially Purkinje cells and glia cells, expressed STAT2 with a strong intensity in CDV-infected dogs and a weak intensity in control dogs. ISG15, MX and PKR proteins were significantly increased at all investigated stages of CDV-induced leukoencephalitis (*p* < 0.001). Perivascular lymphocytes expressed MX and PKR proteins but not ISG15. MX proteins were also detected in endothelial cells, neurons and glial cells of CDV-infected dogs. In control tissue, only some neurons and endothelial cells showed a mild MX expression. Furthermore, glial cells and neurons, especially Purkinje cells and some endothelial cells, were positive for PKR in CDV-infected dogs. In control animals, PKR proteins were only detected in neurons, especially Purkinje cells. ISG15 proteins were detected in neurons, endothelial cells and glial cells of CDV-infected dogs, whereas control animals only showed a weak ISG15 protein expression in neurons and endothelial cells. A significant up-regulation of OAS proteins was found in acute (*p* = 0.008), subacute (*p* < 0.001) and chronic lesions (*p* = 0.01) but not in CDV-infected tissue without histological lesions (group 2; *p* = 0.09). In CDV-infected dogs, OAS proteins were detected in perivascular lymphocytes, endothelial cells, glial cells and neurons. In control animals, only neurons and endothelial cells expressed OAS proteins.

### 2.4. Correlation Analysis

Spearman`s rank correlation coefficients demonstrated a moderate correlation between CDV antigen and STAT1, ISG15, MX and PKR protein expression (Table 3). CDV antigen correlated only weakly with IRF3 and STAT2 and did not correlate with IRF7 protein expression. IRF3 also correlated weakly with STAT1, OAS and PKR. Moreover, there was a weak to moderate correlation between STAT1, ISG15, MX, OAS and PKR, a moderate correlation between IRF7 and ISG15, and a strong correlation between ISG15 and MX (Table 3).

### 2.5. Immunofluorescence

**Figure 5:**
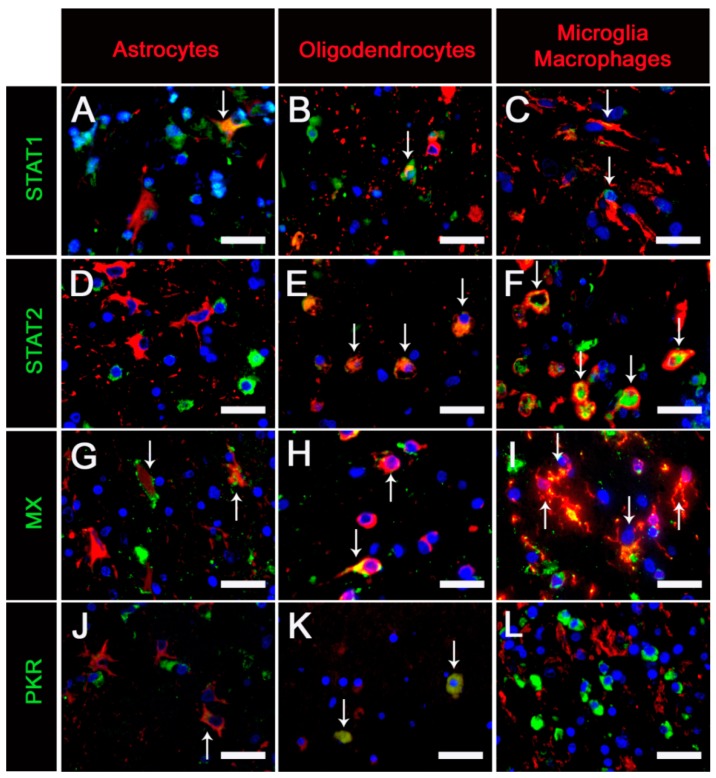
STAT1, STAT2, MX and PKR protein expression of GFAP^+^ astrocytes, NogoA^+^ oligodendrocytes, and IBA1^+^ microglia/macrophages in cerebellar lesions of canine distemper virus-infected dogs. (**A**–**C**) STAT1 is expressed by astrocytes, oligodendrocytes and microglia/macrophages. (**D**–**F**) STAT2 was detected in oligodendrocytes and activated microglia/macrophages (gitter cells). (**G**–**I**) MX was found in astrocytes, oligodendrocytes and microglia/macrophages. (**J**–**L**) PKR is expressed by astrocytes and oligodendrocytes but not by microglia/macrophages. The majority of PKR^+^ cells in L are most likely lymphocytes. Immunofluorescence double-staining using bisbenzimide as nuclear counterstaining. Bars, 50 µm.

Double staining with cellular markers revealed a STAT1 expression in NogoA^+^ oligodendrocytes, GFAP^+^ astrocytes and IBA1^+^ microglia/macrophages, which was limited to histological lesions. STAT2 was detected in NogoA^+^ oligodendrocytes located in white matter lesions and also in normal-appearing white matter. STAT2 was also found in activated IBA1^+^ microglia/macrophages (gitter cells), which were present in subacute and chronic white matter lesions. MX was expressed by intralesional GFAP^+^ astrocytes, NogoA^+^ oligodendrocytes and IBA1^+^ microglia/macrophages. PKR was also found in intralesional GFAP^+^ astrocytes and some NogoA^+^ oligodendrocytes but not in IBA1^+^ microglia/macrophages (Figure 5).

**Figure 6:**
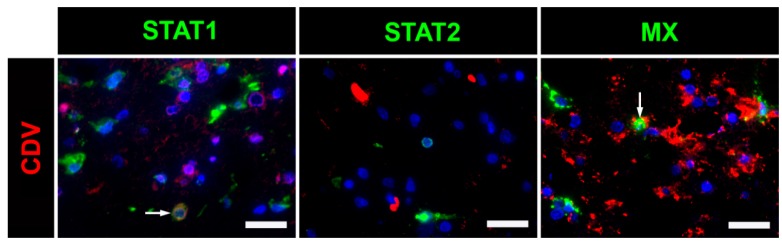
STAT1, STAT2 and MX protein expression in cerebellar lesions of canine distemper virus (CDV)-infected dogs. A co-expression of STAT1 and CDV, as well as MX and CDV, was present in a few cells (arrows), whereas CDV-infected cells expressing STAT2 were nearly absent. Immunofluorescence double-staining using bisbenzimide as nuclear counterstaining. Bars, 50 µm.

Immunofluorescence double-staining was also used to determine the infection status of cells expressing STAT1, STAT2 and MX. A low number of CDV-infected cells expressed STAT1 and MX. However, CDV-infected cells expressing STAT2 were nearly absent (Figure 6).

## 3. Discussion

The present study gives an overview of the protein expression of selected IFN-I pathway members and compares it with existing microarray data. Previous studies often investigated ISG expression in cell cultures, whereas studies on the protein expression of ISGs in a naturally occurring animal disease are rare [48,49,50,51,52]. IRF3 plays, together with the closely related IRF7, an important role in the IFN-I signaling cascade and the control of viral infections [53]. Ysebrant de Lendonck et al. (2014) reported that IRF3 is constitutively expressed in most tissues and cell types [54]. Similarly, in the present study, IRF3 was expressed in most cell types present in CDV-induced cerebellar lesions as well as in control tissue (endothelial cells, neurons, glial cells and perivascular inflammatory cells). This strong constitutive expression of IRF3 might also explain that the present microarray analysis and immunohistochemical investigation did not detect changes in the gene or protein expression of this transcription factor in acute, subacute or chronic cerebellar lesions. Nonetheless, there was a significant increase in IRF3 protein expression in CDV-infected areas lacking histological lesions (group 2) compared to controls demonstrating an early activation of IFN-I signaling in the disease process. IRF3 interacts with other transcription factors, coactivators and repressors including other IRFs and NF-κB and thereby regulates T cell differentiation into Th1, Th2 and Th17 cells as well as induces apoptosis during viral infections [54]. Thus, IRF3 most likely modulates adaptive immune responses at all stages of CDV-induced cerebellar lesions due to its action as a signaling platform, which IRF3 can fulfill without changes in expression.

Unlike IRF3, IRF7 is reported to be expressed only at low levels in most cell types and has to be continuously produced due to its short half-life time of 0.5–1 h [55,56]. Nevertheless, IRF7 protein was present in neurons of the granular layer and individual glial cells, even in control cerebellum, showing low constitutive expression of this transcription factor in the canine CNS. OASL1-knockout mice studies showed that OASL1 inhibits IRF7 translation and thereby negatively regulates IFN-I production during acute and chronic viral infections [57,58]. The present study detected a strong increase in OASL1 mRNA transcripts in acute, subacute and chronic lesions, which might affect IRF7 expression. Nonetheless, there was a strong up-regulation in IRF7 gene and protein expression in cerebellar lesions, which was most pronounced at later stages of CDV-induced leukoencephalitis. This continuous increase in IRF7 expression is probably caused by the positive feedback mechanism of IRF7 and indicates that the IFN-I signaling pathway contributes to lesion progression [56,59,60,61].

STAT1 proteins can form STAT1/STAT2 heterodimers and STAT1/STAT1 homodimers to induce transcription of ISGs and pro-inflammatory cytokines after IFN-I and IFNγ stimulation, respectively [40]. Svitek et al. (2014) demonstrated that CDV has to block STAT2 signaling for immune evasion in ferrets, whereas inhibition of STAT1 does not contribute to CDV virulence [45]. The present study demonstrated a strong increase in STAT1 mRNA transcripts and proteins in the CDV-infected cerebellum (groups 2–5), whereas control animals expressed only minimal amounts of STAT1. STAT2 proteins were constitutively expressed in CDV-infected and control tissue and only weakly up-regulated in a chronic lesion, but microarray analysis revealed a moderate increase in STAT2 mRNA transcripts also in acute and subacute lesions. However, the antibody used to detect STAT1 only binds to phosphorylated and thus activated proteins (p84/p91), whereas the anti-STAT2 antibody recognizes activated and non-activated proteins. This difference explains the detection of less prominent changes in STAT2 compared to STAT1 protein expression using immunohistochemistry.

Interestingly, both transcription factors were found in neurons, oligodendrocytes and activated microglia/macrophages, whereas astrocytes, endothelial cells and inflammatory cells only expressed STAT1 but not STAT2. This observation suggests that the canonical IFN-I pathway with STAT1/STAT2 heterodimer formation only plays a minor role in the latter cells and non-canonical JAK-STAT signaling via STAT1/STAT1 homodimers or other pathways might mediate ISG expression [40]. Moreover, the present analysis of the previously published microarray did not detect an increase in IFN-I expression in the CDV-infected cerebellum, which is most likely caused by the non-structural V protein of CDV interfering with MDA5-dependent virus detection [45]. Consequently, the IFN-I response and the down-stream JAK-STAT signaling cascade are blocked during canine distemper encephalitis. Nevertheless, the present study demonstrated a strong increase in ISG gene and protein expression, which seems to be mediated by IFN-I-independent pathways. Similarly, a recent microarray study of mice infected by Theiler’s murine encephalomyelitis virus (TMEV) described a strong ISG expression in demyelinating spinal cord lesions despite low IFN-I levels [62]. ISG15 expression can directly be induced by IRF1 (formerly called ISGF2), which is a transcription factor involved in anti-viral and anti-bacterial immune responses, T cell and NK cell differentiation, MHC class I and II expression and induction of apoptosis [55,63,64]. Correspondingly, the present canine study demonstrated a strong up-regulation of IRF1 and several MHC genes at all stages of CDV-induced cerebellar lesions. DLA-88 was particularly increased in acute and subacute distemper lesions (fold change up to 135,55). Most importantly, this highly polymorphic MHC class I protein can present peptides derived from CDV hemagglutinin, large polymerase, matrix, nucleocapsid, and V proteins [65,66]. IRF7 is also described as inducing ISG transcription in the absence of IFN-I [67]. Consequently, IRF1- and IRF7-dependent pathways presumably mediate a major part of ISG expression in the CDV-infected cerebellum, counteracting common viral strategies, which modulate or inhibit the IFN-I response [67,68].

ISG15 showed the strongest increase of all investigated genes, with a fold change of almost 1000 in subacute lesions. In addition, a weak ISG15 immunoreactivity was detected in neurons and endothelial cells of uninfected canine cerebella, confirming previous descriptions of a low constitutive ISG15 expression [69]. ISG15 represents an ubiquitin-like protein, which binds to over 150 cellular proteins involved in immune regulation, including members of the IFN-I, NF-κB and JNK pathways [69,70]. This process known as ISGylation can activate or inhibit the respective signaling cascades in a species-specific manner [71]. Murine ISG15 targets RIG1, which reduces type I interferon promoter activity and NF-κB responses [72]. In contrast, human ISG15 can bind to IRF3 preventing its degradation by the proteasome and thereby increasing the IFN-I response [73]. Nevertheless, humans born with inactivating ISG15 mutations are highly susceptible to mycobacterial and autoinflammatory diseases but not to viruses, this underlining the complexity of ISG15-mediated effects [71,74]. The susceptibility of ISG15-deficient patients to environmental mycobacteria is probably related to the cytokine-like functions of secreted ISG15, which acts in synergy with IL12 to induce IFNγ production by T cells or NK cells, thus enhancing the antimycobacterial activity of macrophages [70,71,75,76]. Only a few studies have investigated the functions of ISG15 in dogs, indicating an antiviral activity of this protein in canine cells, which might be mediated by binding ISG15 to viral proteins interfering with viral replication [17,69,70,77]. IFNγ can be found in the CSF of dogs with chronic but not early lesions [78,79,80]. Consequently, highly up-regulated ISG15 levels in CDV-induced cerebellar lesions most likely contribute to viral elimination and might modulate the canine immune system.

PKR can be activated by double-stranded RNA and ISGylation, which results in autophosphorylation and subsequently in phosphorylation of the eukaryotic translation initiation factor 2 alpha (eIF2α), thereby down-regulating cellular and viral protein synthesis [81,82,83,84]. In addition, the activation of this constitutively expressed virus sensor protein can induce apoptosis, thus ensuring the eradication of infected cells from the tissue [85,86]. The present study demonstrated a strong increase in the expression of phosphorylated, hence activated PKR in the CDV-infected cerebellum. This protein was detected in neurons, intralesional astrocytes and oligodendrocytes as well as perivascular lymphocytes but not in microglia/macrophages. Similarly, TMEV-infected mice showed a strong expression of activated PKR proteins in spinal cord white matter lesions [62]. Nevertheless, these proteins were predominantly expressed by intralesional gitter cells and to a lesser extent by perivascular immune cells, oligodendrocytes and neurons but not by astrocytes in TMEV-infected mice. Similarly, in mice suffering from experimental allergic encephalomyelitis, another mouse model of human MS, activated PKR proteins were found in oligodendrocytes and neurons as well as in intralesional T cells and macrophages [87]. These observations suggest a so far undescribed species-specific difference in PKR expression and/or activation of murine and canine astrocytes and microglia/macrophages.

Similar to PKR, OAS proteins are constitutively expressed in many tissues (spleen, lung, liver, thymus, small intestine, brain) and bind double-stranded RNA resulting in their activation [88,89,90]. Activated OAS proteins then induce the formation of 2′-5′-oligoadenylates from ATP molecules, which trigger the ubiquitously expressed endoribonuclease RNase L [91]. This enzyme can degrade cellular and viral RNA and thereby inhibit viral protein synthesis [91,92,93]. The production of small RNA cleavage products by RNase L again initiates IFN-I production, creating a positive feedback mechanism in antiviral defense [93,94]. RNase L has also been described as a potent inducer of apoptosis in fibroblasts [95]. In addition, Kristiansen et al. (2010) suggested that OAS proteins produced by virus-infected cells can be released into the extracellular space, where it acts as an RNase L-independent paracrine antiviral agent to protect neighboring cells from infection [96]. The present study revealed a strong increase in OAS gene and protein expression in acute, subacute and chronic cerebellar lesions, which most likely contributes to the restriction of viral replication. OAS proteins were detected in neurons and endothelial cells as well as in intralesional glial cells and perivascular inflammatory cells. Previous studies demonstrated that the OAS-RNase L pathway is particularly important in murine macrophages and inhibits the replication of *Picornaviridae* including TMEV [93,97,98,99,100,101]. Interestingly, the OAS activity in canine serum is 10- to 100-fold higher than in cats, rabbits and guinea pigs, indicating a prominent role of OAS proteins in the canine humoral immune system [46]. However, the exact role of RNase L-dependent and independent functions of OAS proteins in the pathogenesis of CDV-induced leukoencephalitis remains to be determined.

The present study showed a significant increase in the expression of MX proteins during all stages of canine distemper leukoencephalitis, which was even found in CDV-infected areas without histological lesions. The presence of MX proteins in neurons and endothelial cells of the normal canine brain demonstrates a low but constitutive expression of this antiviral protein, whose expression was described to be strictly dependent on IFN-I [102]. Similarly, Porter et al. (2006) found a strong MX expression in astrocytes, macrophages/microglia and neurons in CDV-infected brain tissue [44]. Endothelial cells were also described to be immunopositive for MX by Porter et al. (2006), but this staining was interpreted as non-specific [44]. However, strong expression of MX proteins in canine distemper lesions most likely impedes viral replication and, similar to increased ISG15, PKR and OAS levels, contributes to the typical decrease in viral antigen observed during the progression of the disease [2,4,103]. Moreover, the low number of CDV-infected cells expressing STAT1, STAT2 and MX indicates that the expression of these type I IFN pathway members is not directly induced by virus infection.

The strong expression of STAT1, STAT2 and downstream ISG proteins in oligodendrocytes and neurons is probably related to their well-known restricted CDV infection, which is characterized by the presence of CDV nucleic acid sequences and lack of viral antigen [4]. In contrast, astrocytes did not express STAT2 proteins, which favors viral replication in these cells due to the blocking of the canonical JAK-STAT pathway. The cell-type specific differences in the activation of the IFN-I pathway might also explain the fact that up to 95% of all CDV-infected cells within acute lesions are astrocytes [104], whereas oligodendrocytes hardly express CDV protein [105]. However, the restriction of virus replication in neurons and oligodendrocytes does not prevent axonal pathology and massive down-regulation of myelin gene expression (oligodendrocyte dystrophy) [105,106]. Both processes contribute to the initiation of the demyelination process, whereas later stages of demyelination are mainly mediated by so-called bystander mechanisms [2,4,47,107].

This bystander demyelination results from the activation of macrophages/microglia, which is characterized by an increased phagocytic activity, oxygen radical production and expression of MHC class II molecules and the release of myelin damaging enzymes such as matrix-metalloproteinases (MMPs) [2,4,108,109,110]. The progressive development of demyelination is driven by a strong continuous expression of IL1, IL6, IL8, IL12 and TNFα promoting innate and Th1-biased immune responses [2,4,80,111,112,113]. Interestingly, recent studies demonstrated that the IFN-I pathway is critically involved in the differentiation and activation of immune cells as well as the expression of MMPs and pro-inflammatory cytokines [55,114,115,116,117,118,119,120]. IFN-I can modulate the phenotype of microglia, regulate phagocytosis and affect blood-brain barrier integrity [115]. IRF1 is required for NK cell development and differentiation of CD8^+^ T cells promotes the differentiation of Th1 cells via transcriptional control of IL12p40, inhibits TNFα-induced MMP9 expression and contributes to the activation of the NLRP3 inflammasome [55,114,116,117]. IRF3 stimulates the transcription of IL6, IL8 and MMPs through interaction with c-Jun and the AP-1 promoter site [118,119,120]. IRF7 regulates the expression of IL6 through stabilization of IL6 mRNA and induces the transcription of CXCL10, a chemokine for macrophages, T cells and NK cells [118]. Moreover, studies of monogenic autoinflammatory CNS diseases demonstrated that they can be caused by an uncontrolled up-regulation of IFN-I signaling and JAK inhibition may be a successful therapeutic strategy in patients with these type I interferonopathies [121,122,123]. Consequently, the strong activation of the IFN-I signaling pathway in infiltrating immune and resident CNS cells including activated microglia/macrophages most likely contributes to immunopathological mechanisms aggravating CDV-triggered demyelination.

## 4. Materials and Methods

### 4.1. Animals

This study was conducted in accordance with the German Animal Welfare Act. All animals used in this study were dead at the time of submission for necropsy in order to investigate the causes of death and disease. Consequently, the authors confirm that this study does not contain data obtained from animal experiments and no animals were infected or sacrificed for the purpose of this retrospective pathological case-control study. Moreover, all dog owners provided written consent for the dogs’ tissues to be collected and used for research purposes.

Paraffin-embedded cerebellar tissue of 15 naturally CDV-infected dogs and six control dogs without any CNS lesion was collected from the archive of the Department of Pathology of the Veterinary University of Hannover, Germany and used for histological analysis and immunostainings. Anamnestic data of the investigated dogs are summarized in Table S2.

### 4.2. Histology and Classification of Cerebellar Lesions

Serial two-micrometer paraffin sections were stained with hematoxylin and eosin (HE) or Luxol fast blue (LFB) or used for immunohistochemistry. The lesions in the CDV-infected dogs were categorized with HE- and LFB-staining as previously described [47,106,124]. Briefly, randomly selected areas in the cerebellar white matter of dogs without any lesions were used as controls (group 1). White matter lesions of CDV-infected dogs were divided into four groups. Group 2 (“antigen without lesion”) included areas with antigen expression but with no lesions in HE- or LFB-stainings. Group 3 lesions (“acute”) were characterized by vacuolation in the HE staining and absence of demyelination in the LFB-staining. Group 4 (“subacute”) included lesions with demyelination but with no inflammatory infiltrates in the HE-staining. Group 5 (“chronic”) contained demyelinated lesions with variable numbers of inflammatory cells in the perivascular area or diffusely distributed ones in the parenchyma.

### 4.3. Transcriptional Analysis of Interferon-stimulated Genes

An MIAME-compliant expression raw data set of a previously published microarray study upon CDV-induced demyelinating leukoencephalitis performed on GeneChip canine genome 2.0 arrays (Affymetrix Inc., Santa Clara, USA) was accessed via the ArrayExpress database (accession number: E-MEXP-3917; http://www.ebi.ac.uk/arrayexpress) [47]. RNA used for the original microarray study was isolated from frozen cerebellar specimens of 12 control dogs and 14 CDV-infected dogs, which were grouped using a classification system similar to the present study (control; acute; subacute; chronic) [47]. Fold changes in this data set were calculated as the ratio of the inverse-transformed arithmetic means of the log2-transformed expression values between the respective groups. Downregulations are given as negative reciprocal values [47]. In order to perform a bottom-up analysis of transcriptional changes in CDV-induced lesions, 110 candidate genes including 12 pattern recognition receptors (PRRs), ten IFN regulatory factors, eight type I/II IFNs, five type I/II/III receptors, 17 signal transducers, 46 IFN-dependent antiviral effectors and 12 MHC class I/II genes were manually extracted from peer-reviewed published literature (Table S1) [12,30,62,125,126]. If necessary, murine and human genes were converted into orthologous canine gene symbols using the MADGene web tool (http://cardioserve.nantes.inserm.fr/madtools/madgene/) [127]. The expression values of these genes were evaluated for significant differences between the respective groups employing independent pairwise Mann-Whitney U tests (see statistical analysis).

### 4.4. Immunohistochemistry

For immunohistochemistry, sections were dewaxed, rehydrated and incubated in ethanol with 0.5% hydrogen peroxide for 30 min to block endogenous peroxidase. Except for STAT1, sections were pretreated by boiling them in 10 mM sodium citrate buffer (pH 6) for 20 min in a microwave oven (800 W). Sections were blocked with phosphate-buffered saline (PBS) containing 20% goat serum for 30 min at room temperature and incubated with rabbit polyclonal anti-phosphorylated STAT1 (p84/p91, sc-346, Santa Cruz Biotechnology Inc., Santa Cruz, CA, USA, 1:400), anti-OAS (sc-98424, Santa Cruz, 1:600) and anti-ISG15 (sc-50366, Santa Cruz, 1:600), rabbit monoclonal anti-IRF3 (ab68481, Abcam Inc., Cambridge, MA, USA, 1:2000), anti-IRF7 (ab109255, Abcam, 1:8000) and anti-phosphorylated PKR (phospho T446, ab32036, Abcam, 1:600) as well as mouse monoclonal anti-CDV (D110; kindly provided by Prof. Dr. A. Zurbriggen, University of Bern, Bern, Switzerland, 1:100), anti-STAT2 (sc-1668, Santa Cruz, 1:100) and anti-MX (M143, kindly provided by Prof. Dr. Haller and PD. Dr. Kochs, University Medical Center Freiburg, Freiburg, Germany, 1:1000) antibodies overnight at 4 °C. Sections incubated with normal rabbit serum (R4505, Sigma-Aldrich Chemie GmbH, Munich, Germany) or IgG-containing ascites of Balb/C mice instead of primary antibodies served as negative controls. Biotinylated goat-anti-rabbit IgG (BA-1000) or goat-anti-mouse IgG (BA-9200), diluted 1:200 (Vector Laboratories Inc., Burlingame, CA, USA), were used as secondary antibodies. Immunolabeling was visualized by using the avidin–biotin–peroxidase complex method (PK6100, Vector Laboratories) and the chromogen 3,3′-diaminobenzidine-tetrahydrochloride with 0.05% hydrogen peroxide. Finally, sections were slightly counterstained with Mayer’s hematoxylin.

Every immunohistochemically stained slide was photographed with a digital microscope (HS All-in-one Fluorescence Microscope BZ-9000 Generation II, BIOREVO, KEYENCE Deutschland GmbH, Neu-Isenburg, Germany) and afterwards analyzed using analysis software (analySIS 3.1 software package, Soft Imaging System GmbH, Münster, Germany). Percentage of immunopositive areas are shown as Box-and-Whisker plots with median, minimum and maximum in a logarithmic scale.

### 4.5. Immunofluorescence Double-Labeling

To determine the cellular origin of selected members of the JAK-STAT signaling pathway (STAT1, STAT2) and downstream ISGs (MX and PKR), immunofluorescence double-labeling was performed using markers for oligodendrocytes (NogoA), astrocytes (GFAP) and microglia/macrophages (IBA1). Moreover, double-staining was used to investigate the infection status of cells expressing STAT1, STAT2 and MX. Immunofluorescence was performed on selected sections of subacute and chronic lesions due to the strong expression of the investigated markers in the microarray analysis and immunohistochemical evaluation.

Dewaxing, rehydration, pretreatment and blocking of non-specific binding with 20% goat serum or 20% horse serum was performed as described for immunohistochemistry without blocking the endogenous peroxidase. Sections were co-incubated with antibodies directed against phosphorylated STAT1 (sc-346; 1:100), STAT2 (sc-1668; 1:50), MX (M143; 1:100) or phosphorylated PKR (ab32036; 1:50) and antibodies directed against GFAP (goat polyclonal, ab53554, Abcam, 1:200 or rabbit polyclonal, Dakocytomation GmbH, Hamburg, Germany, 1:1000), NogoA (C-4, mouse monoclonal, sc-271878, Santa Cruz, 1:200 or rabbit polyclonal, Millipore/Chemicon, Catalog-No. AB5664P, 1:500), IBA1 (mouse monoclonal, ab15690, Abcam, 1:300 or rabbit polyclonal, ThermoFisher Scientific, Schwerte, Germany, Catalog-No. PA5-27436, 1:500) or CDV (D110, mouse monoclonal, 1:100 or #25, kindly provided by C. Örvell, Karolinska University Hospital, Stockholm, Sweden, rabbit polyclonal, 1:2000) overnight at 4 °C. Negative controls were included in accordance with the immunohistochemical investigation. Subsequently, sections were incubated with Cy2- and Cy3-conjugated secondary antibodies (goat anti-rabbit, 111-165-144; goat anti-mouse, 115-545-003 or 115-165-166; donkey anti-goat, 705-165-147; Jackson ImmunoResearch Europe Ltd., Cambridge, UK; goat anti-rabbit, A-11034; Invitrogen/Thermo Fisher; donkey anti-rabbit, ab150073; Abcam; 1:200) for 45 min at room temperature in the dark. Nuclei were visualized by 0.01% bisbenzimide (H33258, Sigma-Aldrich) and sections were mounted with mounting medium (Shandon Immu-Mount, ThermoFisher Scientific, Catalog-No. 9990402, USA). Sections were photographed with a digital microscope (HS All-in-one Fluorescence Microscope BZ-9000 Generation II, BIOREVO, KEYENCE Deutschland GmbH, Neu-Isenburg, Germany).

### 4.6. Statistical Analysis

Microarray data were analyzed with non-parametric Mann-Whitney U tests (Prism 6, GraphPad Software Ltd., La Jolla, CA, USA), comparing each group of CDV-infected dogs with controls. Immunohistochemically data were analyzed using Kruskal-Wallis tests and Dunn’s multiple comparison tests comparing groups 2–5 (CDV-infected) with group 1 (control). Spearman`s rank coefficients were calculated to evaluate the correlation of CDV antigen and ISG protein expression. Statistical significance was designated as *p* < 0.05.

## 5. Conclusions

The IFN-I pathway is activated in the early stages of CDV-induced leukoencephalitis, leading to the expression of various ISGs, which restrict viral replication particularly, in neurons and oligodendrocytes. In contrast, the lack of STAT2 expression in astrocytes and subsequent suppression of canonical JAK-STAT signaling might explain their high infection rate in cerebellar lesions. The up-regulation of ISGs seems to be partly mediated by IRF1- and IRF7-dependent pathways counteracting the inhibition of the IFN-I response by CDV. However, the strong activation of the IFN-I signaling cascade, especially in activated microglia/macrophages, probably contributes to immune-mediated mechanisms of demyelination in later stages of the disease. These results encourage further research to elucidate the regulation of IFN-I signaling and its contribution to demyelinating CNS diseases in dogs and humans in order to develop novel treatment strategies targeting specific IFN-I pathway members.

## Figures and Tables

**Table 1:** Transcriptional changes of type I interferon signaling pathway members in canine distemper virus-infected compared to control dogs.

Gene Symbol	Acute	Subacute	Chronic
**Pattern recognition receptors**			
**Eif2ak2 (PKR)**	17.71 *	15.62 *	6.90 *
Ifih1 (MDA5)	22.26 *	33.76 *	20.76 *
Tlr2	2.10 *	2.84 *	4.52 *
Tlr3	4.76 *	5.87 *	8.00 *
Tlr7	2.87 *	2.83 *	2.60 *
**Interferon regulatory factors**			
Irf1	14.73 *	30.13 *	13.98 *
**Irf3**	1.17	1.23	1.32
Irf5	1.06	1.03	1.71
**Irf7**	45.42 *	113.54 *	24.65 *
Nfkb1	1.23	1.59 *	1.39
**Type I interferons**			
Ifna1	1.00	1.00	1.00
Ifnb1	−1.00	1.01	−1.00
**Type I interferon receptors**			
Ifnar1	1.33	1.53 *	−1.14
Ifnar2	1.21	1.52 *	1.25
**Signal transducers**			
Irf9	8.16 *	11.44 *	3.38 *
Jak1	1.12	−1.27	−1.22
Socs1	1.00	1.00	1.00
**Stat1**	12.03 *	19.19 *	7.12 *
**Stat2**	3.21 *	4.57 *	2.63 *
Tyk2	1.00	1.00	1.00
**Interferon-stimulated genes**			
IFI44	69.67 *	78.05 *	23.72 *
Ifi44l	275.63 *	419.93 *	109.23 *
Ifit1 (Isg56)	93.57 *	179.05 *	47.07 *
Ifit2 (Isg54)	39.89 *	77.21 *	25.89 *
**Isg15**	590.52 *	928.35 *	245.71 *
Isg20	4.62 *	8.05 *	2.87
**Mx1**	11.44 *	15.98 *	7.35 *
Mx2	47.68 *	147.59 *	38.43 *
**Oas1**	42.18 *	71.15 *	10.97 *
Oas2	80.24 *	173.57 *	34.76 *
Oasl	44.47 *	118.26 *	33.62 *
Oasl2	4.42 *	8.14 *	2.61 *
Rnasel	5.88 *	9.07 *	3.29 *
**Major histocompatibility genes class I/II**			
DLA-12 (MHC I)	6.72 *	10.64 *	4.94 *
DLA-64 (MHC I)	24.52 *	37.51 *	18.07 *
DLA-79 (MHC I)	10.49 *	36.99 *	9.17 *
DLA-88 (MHC I)	17.87 *	135.55 *	−4.80
DLA-DQA1 (MHC II)	12.19 *	30.97 *	17.87 *
DLA-DQB1 (MHC II)	13.34 *	22.98 *	14.09 *
DLA-DRA (MHC II)	5.27 *	8.34 *	6.78 *
DLA-DRB1 (MHC II)	6.65 *	9.77 *	7.64 *

Shown are fold changes in acute, subacute and chronic lesions. Significant fold change differences are shown in italics and marked with an asterisk (* *p* < 0.05). Bold letters indicate up-regulation higher than 4.0. Legend: DLA: dog leukocyte antigen; Eif2ak2: eukaryotic translation initiation factor 2 alpha kinase 2; Ifi: interferon-induced protein; Ifih: interferon-induced with helicase C domain; Ifit: interferon-induced protein with tetratricopeptide repeats; Ifna: interferon α; Ifnb: interferon β; Ifnar: interferon α receptor; Irf: interferon regulatory factor; Isg: interferon-stimulated gene; Jak: janus kinase; MDA: melanoma differentiation antigen; MHC: major histocompatibility complex; Mx: myxovirus (influenza virus)-resistant protein; Nfkb: nuclear factor of kappa light polypeptide gene enhancer in B cells; OAS: 2′-5′-oligoadenylate synthetase; PKR: protein kinase R; Socs: suppressor of cytokine signaling; Stat: signal transducer and activator of transcription; Rnasel: ribonuclease L; Tlr: toll-like receptor; Tyk: tyrosine kinase.

**Table 2:** Immunohistochemical expression of IRF3, IRF7, STAT1, STAT2, ISG15, MX, PKR and OAS in canine distemper virus (CDV)-infected and control cerebellar tissue.

	IRF3		IRF7		STAT1		STAT2		ISG15		MX		OAS		PKR	
**CDV**	+	-	+	-	+	-	+	-	+	-	+	-	+	-	+	-
**Neurons**	+	+	++	++	+	(+)	++	++	++	+	+++	+	++	++	++	++
**Purkinje cells**	+	(+)	(+)	(+)	+	(+)	++	++	++	+	+++	-	++	++	+++	+++
**Granular cells**	+	+	++	++	-	-	-	-	+	-	+++	-	(+)	(+)	-	-
**Glial cells**	++	++	++	++	+++	+	+++	++	+	-	+++	-	+	-	++	-
**Endothelial cells**	+++	+++	(+)	(+)	+	(+)	-	-	++	(+)	++	(+)	+	+	(+)	-
**Inflammatory cells**	+++	n.a.	-	n.a.	+	n.a.	-	n.a.	-	n.a.	++	n.a.	++	n.a.	+++	n.a.

+++ = 75–100% positive cells; ++ = 30–75% positive cells; + = 10–30% positive cells; (+) = only single cells positive, <10%; - = no positive cells; n.a. = not applicable.

**Table 3:** Spearman’s rank correlation coefficients (*r_s_*) between canine distemper virus (CDV) antigen and STAT1, STAT2, IRF3, IRF7, ISG15, MX, OAS and PKR protein expression in CDV-infected and control cerebellar tissue.

	CDV	IRF3	IRF7	STAT1	STAT2	ISG15	MX	OAS	PKR
**CDV**	.	0.205*	0.026	0.541 *	0.259 *	0.490 *	0.594 *	0.289 *	0.454 *
**IRF3**	0.205 *	.	−0.034	0.162 *	0.034	0.052	−0.063	0.204 *	0.249 *
**IRF7**	0.026	−0.034	.	0.334 *	0.146 *	0.404 *	0.332 *	0.268 *	0.225 *
**STAT1**	0.541 *	0.162*	0.334 *	.	0.357 *	0.442 *	0.586 *	0.404 *	0.436 *
**STAT2**	0.259 *	0.034	0.146 *	0.357 *	.	0.225 *	0.314 *	0.174 *	0.256 *
**ISG15**	0.490 *	0.052	0.404 *	0.442 *	0.225 *	.	0.640 *	0.454 *	0.471 *
**MX**	0.594 *	−0.063	0.332 *	0.586 *	0.314 *	0.640 *	.	0.423 *	0.520 *
**OAS**	0.289 *	0.204 *	0.268 *	0.404 *	0.174 *	0.454 *	0.423 *	.	0.331 *
**PKR**	0.454 *	0.249 *	0.225 *	0.436 *	0.256 *	0.471 *	0.520 *	0.331 *	.

Light green: *r_s_* > 0.4; dark green: *r_s_* > 0.6; * *p* < 0.05.

## Supplementary Materials

Supplementary Materials can be found at https://www.mdpi.com/1422-0067/20/7/1620/s1.
