# Supplementary material for: Interferon-Stimulated Genes—Mediators of the Innate Immune Response during Canine Distemper Virus Infection
Source: Int J Mol Sci. 2019 Apr 1;20(7):1620. doi: 10.3390/ijms20071620 (PMC6480560; doi:10.3390/ijms20071620)
Supplement: Supplementary file 1 [file ijms-20-01620-s001.pdf]

Supplementary Materials

Interferon stimulated genes - mediators of the innate immune response during canine distemper virus infection

Daniela Klotz, Ingo Gerhauser

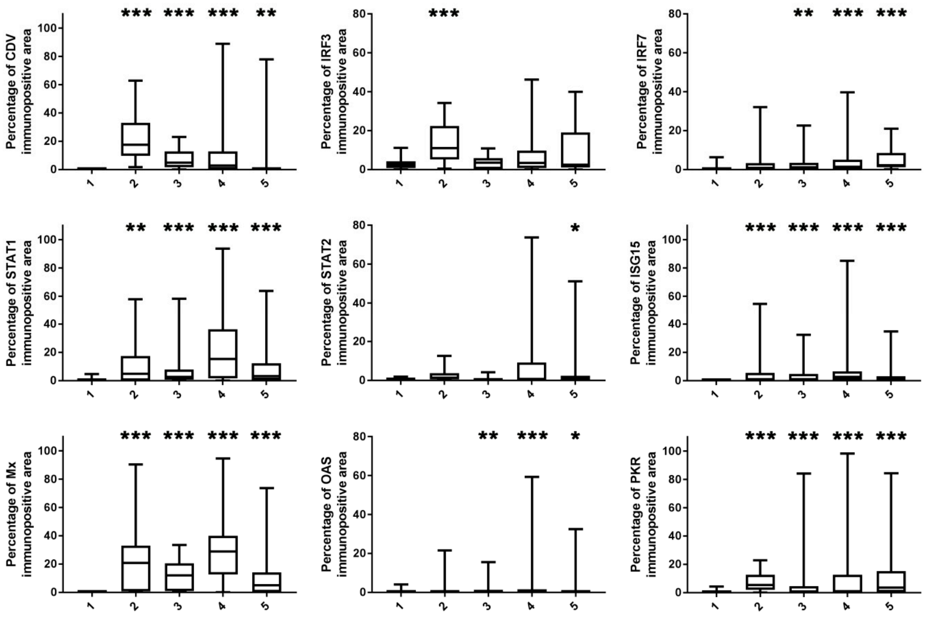

Figure S1. Linear representation of Figure 3: Canine distemper virus (CDV) antigen and IRF3, IRF7, STAT1, STAT2, ISG15, MX, OAS and PKR protein expression in the cerebellar white matter. Group 1 are areas in the white matter of control dogs; Group 2 are foci with CDV antigen expression but without histological lesions; Group 3 are acute CDV lesions; Group 4 are subacute foci without inflammation; Group 5 are chronic CDV lesions with inflammation. Shown are the percentages of immunopositive area using box plots and significant differences between the CDV-infected groups 2-5 and the control group 1 based on Kruskal-Wallis-tests and Dunn's multiple comparison tests. *$P < 0.05$; **$P < 0.01$; ***$P < 0.001$.

Table S1. Transcriptional changes of the type I, II and III IFN signaling pathway in canine distemper virus-infected compared to control dogs.

Probe Set ID	Gene Symbol	Gene Title	Acute		Subacute		Chronic	
			Fold change	*P value*	Fold change	*P value*	Fold change	*P value*
		**Pattern Recognition Receptors**						
Cfa.871.2.S1_s_at	Ddx58 (RIG-I)	DEAD (Asp-Glu-Ala-Asp) box polypeptide 58	39.07*	<0.001	43.99*	<0.001	11.97*	0.004
CfaAffx.10071.1.S1_s_at	Eif2ak2 (PKR)	Eukaryotic translation initiation factor 2-alpha kinase 4	17.71*	<0.001	15.62*	<0.001	6.90*	0.004
CfaAffx.16351.1.S1_s_at	Ifih1 (MDA5)	Interferon induced with helicase C domain 1	22.26*	<0.001	33.76*	<0.001	20.76*	0.004
CfaAffx.24815.1.S1_at	Tlr1	Toll-like receptor 1	3.46*	<0.001	4.54*	<0.001	1.84	0.866
CfaAffx.13248.1.S1_s_at	Tlr2	Toll-like receptor 2	2.10*	0.049	2.84*	0.002	4.52*	0.004
CfaAffx.11983.1.S1_at	Tlr3	Toll-like receptor 3	4.76*	0.001	5.87*	0.001	8.00*	0.004
CfaAffx.601.1.S1_s_at	Tlr4	Toll-like receptor 4	1.80*	0.004	2.24*	0.009	1.84	0.130
CfaAffx.17705.1.S1_at	Tlr5	Toll-like receptor 5	1.05	0.461	1.01	0.186	1.00	0.319
Cfa.12377.1.A1_at	Tlr7	Toll-like receptor 7	2.89*	<0.001	2.83*	0.010	2.60*	0.011

CfaAffx.18172.1.S1_at	Tlr8	Toll-like receptor 8	2.32*	<0.001	4.44*	<0.001	1.64*	0.004
Cfa.824.1.S1_at	Tlr9	Toll-like receptor 9	-1.05	0.266	-1.03	0.890	-1.06	0.134
CfaAffx.24819.1.S1_at	Tlr10	Toll-like receptor 10	1.67	0.077	1.42	0.159	1.73	0.855
**Interferon Regulatory Factors**								
CfaAffx.2202.1.S1_s_at	Irf1	Interferon regulatory factor 1	14.73*	<0.001	30.13*	<0.001	13.98*	0.002
CfaAffx.12486.1.S1_s_at	Irf2	Interferon regulatory factor 2	1.39	0.082	1.53	0.053	1.26	0.233
Cfa.18322.1.S1_at	Irf3	Interferon regulatory factor 3	1.17	0.662	1.23	0.983	1.32	0.752
CfaAffx.14437.1.S1_at	Irf4	Interferon regulatory factor 4	1.00	>0.999	1.00	>0.999	3.00*	0.029
CfaAffx.3326.1.S1_s_at	Irf5	Interferon regulatory factor 5	1.06	0.074	1.03	0.044	1.71	0.200
CfaAffx.18603.1.S1_s_at	Irf6	Interferon regulatory factor 6	1.01	0.838	-1.01	0.838	-1.01	>0.999
CfaAffx.10779.1.S1_at	Irf7	Interferon regulatory factor 7	45.42*	<0.001	113.54*	<0.001	24.65*	0.002
Cfa.9365.1.A1_at	Irf8	Interferon regulatory factor 8	2.00*	0.001	2.02*	0.001	2.74*	0.004
CfaAffx.16794.1.S1_s_at	Nfkb1	Nuclear factor of kappa light polypeptide gene enhancer in B cells 1, p105	1.23	0.104	1.59*	0.010	1.39	0.070
CfaAffx.20636.1.S1_s_at	Rela	v-rel reticuloendotheliosis viral oncogene homolog A (avian)	-1.04	0.878	-1.01	>0.999	-1.23	0.191
**Type I/II/III Interferons**								
CfaAffx.3402.1.S1_at	Ifna1	interferon alpha-1/2-like	1.00	>0.999	1.00	>0.999	1.00	>0.999
CfaAffx.3390.1.S1_at	Ifna2	interferon alpha-2-like	-1.01	0.559	-1.01	0.529	-1.01	>0.999
Cfa.16250.1.S1_x_at	Ifna5°	Interferon alpha 5	1.01	0.294	1.00	>0.999	1.00	>0.999
CfaAffx.3389.1.S1_at	Ifnb1	Interferon beta 1, fibroblast	-1.00	>0.999	1.01	0.441	-1.00	>0.999
CfaAffx.3408.1.S1_at	Ifne	Interferon epsilon	1.00	>0.999	1.00	>0.999	1.00	>0.999
Cfa.3900.1.S1_s_at	Ifng	Interferon gamma	-1.00	>0.999	1.01	0.098	1.02	0.004
CfaAffx.3468.1.S1_at	Ifnk	Interferon kappa	-1.00	>0.999	-1.00	>0.999	-1.00	>0.999
CfaAffx.9324.1.S1_at	Ifnl1	Interferon lambda 1	1.00	>0.999	1.00	>0.999	1.00	>0.999
**Type I/II/III Interferon Receptors**								
Cfa.19476.1.S1_s_at	Ifnar1	interferon alpha/beta receptor 1-like	1.33	0.383	1.53*	0.005	-1.14	0.470
Cfa.10009.1.A1_at	Ifnar2	interferon (alpha, beta and omega) receptor 2	1.21	0.082	1.52*	0.010	1.25	0.136
CfaAffx.1357.1.S1_at	Ifngr1	Interferon gamma receptor 1	1.38	0.064	1.31	0.151	1.42	0.734
Cfa.14847.1.A1_s_at	Ifngr2	Interferon gamma receptor 2-like	1.07	0.678	1.20	0.436	1.51*	0.048
CfaAffx.20153.1.S1_at	Ifnlr1	Interferon lambda receptor 1	1.05	0.294	1.00	>0.999	1.01	0.200
**Signal Transducers**								
CfaAffx.18651.1.S1_at	Irf9	Interferon regulatory factor 9	8.16*	<0.001	11.44*	<0.001	3.38*	0.002
Cfa.15782.2.S1_s_at	Jak1	Janus kinase 1	1.12	0.796	-1.27	0.468	-1.22	0.470
Cfa.16973.1.S1_at	Jak2	Janus kinase 2	1.84	0.796	2.34	0.468	2.17	0.470
CfaAffx.23353.1.S1_at	Jak3	Janus kinase 3	1.00*	0.002	1.00*	<0.001	1.00*	0.009
CfaAffx.28878.1.S1_at	Socs1	Suppressor of cytokine signaling 1	1.00	>0.999	1.00	>0.999	1.00	>0.999
CfaAffx.10255.1.S1_at	Socs2	Suppressor of cytokine signaling 2	-1.03	0.903	-2.50*	0.002	1.20	0.295
Cfa.3905.1.S1_at	Socs3	Suppressor of cytokine signaling 3	1.11	0.006	1.01*	0.020	-1.00	0.40
Cfa.7833.1.A1_s_at	Socs4	Suppressor of cytokine signaling 4	-1.13	0.663	-1.41	0.450	1.33	0.306
Cfa.10850.1.A1_s_at	Socs5	Suppressor of cytokine signaling 5	-1.04	0.603	-1.15	0.145	-1.18	0.154
CfaAffx.25443.1.S1_at	Socs7	Suppressor of cytokine signaling 7	1.06	0.182	1.02	0.367	-1.11*	0.004
Cfa.18084.1.S1_s_at	Stat1	signal transducer and activator of transcription 1-like /// signal transducer and activator of transcription 4	12.03*	<0.001	19.19*	<0.001	7.12*	0.004
CfaAffx.27105.1.S1_s_at	Stat2	Signal transducer and activator of transcription 2	3.21*	<0.001	4.57*	<0.001	2.63*	0.004
CfaAffx.23466.1.S1_s_at	Stat3	signal transducer and activator of transcription 3 (acute-phase response factor)	1.85*	0.001	2.01*	<0.001	1.50*	0.048
Cfa.2683.1.A1_at	Stat5A	signal transducer and activator of transcription 5A	1.20*	0.001	1.42*	<0.001	1.18*	0.009
CfaAffx.23894.1.S1_at	Stat5B	signal transducer and activator of transcription 5B	1.12	0.273	1.37*	0.005	1.16	0.196
Cfa.15552.1.A1_at	Stat6	signal transducer and activator of transcription 6, interleukin-4 induced	-1.05	0.825	1.09	0.397	-1.43*	0.044
CfaAffx.27321.1.S1_s_at	Tyk2	Tyrosine kinase 2	1.00	>0.999	1.00	>0.999	1.00	>0.999
**Interferon-Dependent Antiviral Effectors**								

CfaAffx.26268.1.S1_at	Adar	Adenosine deaminase, RNA-specific	1.44	0.061	_1.92*_	0.001	-1.01	0.820
CfaAffx.2928.1.S1_at	Apobec3h	Apolipoprotein B mRNA editing enzyme, catalytic polypeptide-like 3H	1.05	0.131	1,66	0.067	1.42	0.207
Cfa.6729.1.A1_at	Cldn4	Claudin 4	-1.17	0.184	-1.01	0.666	-1.07	0.310
Cfa.16947.1.A1_at	Ch25h	Cholesterol 25-hydroxylase	**_4.12*_**	<0.001	**_4.68*_**	0.001	2.48	0.101
CfaAffx.22274.1.S1_x_at	Ddit4	DNA-damage-inducible transcript 4	_1.54*_	0.046	1,67	0.050	1.22	0.222
Cfa.17424.1.S1_s_at	Ddx17	DEAD (Asp-Glu-Ala-Asp) box polypeptide 17	-1.13	0.202	-1.17	0.127	-1.13	0.664
CfaAffx.14223.1.S1_at	Ddx60	DEAD (Asp-Glu-Ala-Asp) box polypeptide 60	**_25.50*_**	<0.001	**_39.85*_**	<0.001	**_31.32*_**	0.002
CfaAffx.30940.1.S1_s_at	Gbp1	Guanylate binding protein 1	**_30.46*_**	<0.001	**_88.40*_**	<0.001	**_55.46*_**	0.002
CfaAffx.14369.1.S1_at	Hpse	Heparanase	1.01	0.932	1.26	0.767	_1.28*_	0.037
Cfa.20456.1.S1_s_at	Ifi6	interferon, alpha-inducible protein 6	**_4.18*_**	<0.001	**_6.45*_**	<0.001	_2.57*_	0.004
CfaAffx.18191.1.S1_s_at	IFi16	interferon, gamma-inducible protein 16	**_16.05*_**	<0.001	**_19.31*_**	<0.001	**_15.43*_**	0.004
Cfa.16440.1.S1_at	Ifi35	Interferon-induced protein 35	**_9.72*_**	<0.001	**_17.19*_**	<0.001	**_9.32*_**	0.004
Cfa.18904.1.S1_s_at	IFI44	Interferon-induced protein 44	**_69.67*_**	<0.001	**_78.05*_**	<0.001	**_23.72*_**	0.004
CfaAffx.31150.1.S1_at	Ifi44l	Interferon-induced protein 44 like	**_275.63*_**	<0.001	**_419.93*_**	<0.001	**_109.23*_**	0.004
CfaAffx.15121.1.S1_at	Ifit1 (Isg56)	Interferon-induced protein with tetratricopeptide repeats 1	**_93.57*_**	<0.001	**_179.05*_**	<0.001	**_47.07*_**	0.004
Cfa.9758.1.A1_at	Ifit2 (Isg54)	Interferon-induced protein with tetratricopeptide repeats 2	**_39.89*_**	<0.001	**_77.21*_**	<0.001	**_25.89*_**	0.004
Cfa.19536.1.S1_at	Ifit5	interferon-induced protein with tetratricopeptide repeats 5	_2.75*_	<0.001	**_4.41*_**	<0.001	_1.91*_	0.004
CfaAffx.15732.1.S1_at	Ifitm10	Interferon induced transmembrane protein 10	-1.05	0.954	-1.19	0.145	-1.13	0.391
Cfa.10757.1.S1_at	Isg15	ISG15 ubiquitin-like modifier	**_590.52*_**	<0.001	**_928.35*_**	<0.001	**_245.71*_**	0.004
CfaAffx.17917.1.S1_at	Isg20	Interferon-stimulated protein	**_4.62*_**	<0.001	**_8.05*_**	<0.001	2.87	0.165
CfaAffx.21280.1.S1_at	Map3k14 (Nik)	Mitogen-activated protein kinase kinase kinase 14	1.00	>0.999	1.00	>0.999	1.00	>0.999
CfaAffx.20794.1.S1_at	Mov10	Moloney leukemia virus 10	_3.57*_	<0.001	**_5.21*_**	<0.001	_3.25*_	0.004
CfaAffx.15920.1.S1_s_at	Mx1	Myxovirus (influenza virus) resistance 1	**_11.44*_**	<0.001	**_15.98*_**	<0.001	**_7.35*_**	0.004
Cfa.3609.1.S1_s_at	Mx2	Myxovirus (influenza virus) resistance 2	**_47.68*_**	<0.001	**_147.59*_**	<0.001	**_38.43*_**	0.004
CfaAffx.6906.1.S1_at	Nampt (Pbef1)	Nicotinamide phosphoribosyltransferase	_1.36*_	0.027	_1.70*_	0.002	1.64	0.101
Cfa.20355.1.S1_at	NT5C3A	5'-nucleotidase, cytosolic IIIA	_2.08*_	<0.001	_2.93*_	<0.001	_1.43*_	0.009
Cfa.20525.1.S1_at	NT5C3B	5'-nucleotidase, cytosolic IIIB	_-1.10*_	0.036	_-1.20*_	0.041	-1.12	0.255
Cfa.9330.1.A1_at	Oas1	2'-5' oligoadenylate synthetase 1	**_42.18*_**	<0.001	**_71.15*_**	<0.001	**_10.97*_**	0.004
CfaAffx.14116.1.S1_at	Oas2	2'-5' oligoadenylate synthetase 2	**_80.24*_**	<0.001	**_173.57*_**	<0.001	**_34.76*_**	0.004
CfaAffx.14109.1.S1_s_at	Oas3	2'-5' oligoadenylate synthetase 3	_1.27*_	0.004	_1.51*_	0.002	1.16	0.319
CfaAffx.16440.1.S1_at	Oasl	2'-5' oligoadenylate synthetase-like	**_44.47*_**	<0.001	**_118.26*_**	<0.001	**_33.62*_**	0.002
CfaAffx.16447.1.S1_s_at	Oasl2	2'-5' oligoadenylate synthetase-like 2	**_4.42*_**	<0.001	**_8.14*_**	<0.001	_2.61*_	0.009
CfaAffx.9374.1.S1_at	P2ry6	Pyrimidinergic receptor P2Y, G-protein coupled, 6	1.35	0.382	1.02	0.872	2.02	0.519
Cfa.14949.1.A1_s_at	PHF15	PHD finger protein 15	_1.73*_	0.030	_2.31*_	<0.001	_2.13*_	0.022
CfaAffx.27337.1.S1_at	Pml (Trim19)	Promyelocytic leukemia	-1.04	0.778	1.07	0.731	1.08	0.275
CfaAffx.20321.1.S1_at	Rnasel	Ribonuclease L (2', 5'-oligoisoadenylate synthetase-dependent)	**_5.88*_**	<0.001	**_9.07*_**	<0.001	_3.29*_	0.004
CfaAffx.5863.1.S1_at	Rsad2 (Viperin)	Radical S-adenosyl methionine domain containing 2	**_10.80*_**	<0.001	**_25.01*_**	<0.001	_3.17*_	0.002
CfaAffx.21443.1.S1_at	Rtp4	Receptor transporter protein 4	**_24.43*_**	<0.001	**_27.91*_**	<0.001	**_9.71*_**	0.004
Cfa.9597.1.S1_at	Samhd1	SAM domain and HD domain, 1	_1.40*_	<0.001	_1.90*_	0.018	1.16	0.136
CfaAffx.25103.1.S1_at	Slc15a3	Solute carrier family 15, member 3	1.00	0.294	1.00	0.333	1.00	0.200

CfaAffx.13398.1.S1_at	Slc25a28	Solute carrier family 25, member 28	-1.11	0.109	1.09	0.485	-1.19	0.152
CfaAffx.29066.1.S1_at	Ssbp3	Single-stranded DNA binding protein 3	-1.39	0.061	-1.61	0.524	-1.81	0.119
CfaAffx.833.1.S1_at	Trex1 (Atrip)	Three prime repair exonuclease 1 (ATR interacting protein)	1.28	0.109	_1.83*_	0.005	1.14	0.468
CfaAffx.26603.1.S1_s_at	Trim25	Tripartite motif-containing 25	1.00	0.191	_1.14*_	0.005	1.08	0.200
Cfa.18920.1.S1_s_at	Sun2 (Unc84b)	Sad1 and UNC84 domain containing 2	1.10	0.524	_-1.12*_	0.050	1.06	0.574
CfaAffx.20922.1.S1_at	Zbtb16 (Plzf)	zinc finger and BTB domain containing 16	1.57	0.104	1.69	0.103	1.72	0.180
**Major Histocompatibility Genes Class I/II**								
Cfa.1333.3.S1_a_at	Cd74	CD74 antigen (invariant polypeptide of major histocompatibility complex, class II antigen-associated)	**_7.30*_**	<0.001	**_9.89*_**	<0.001	**_6.06*_**	0.009
Cfa.20996.1.S1_at	DLA-12 (MHC I)	MHC class I DLA-12	**_6.72*_**	<0.001	**_10.64*_**	<0.001	**_4.94*_**	0.004
CfaAffx.1704.1.S1_s_at	DLA-64 (MHC I)	MHC class I DLA-64	**_24.52*_**	<0.001	**_37.51*_**	<0001	**_18.07*_**	0.004
Cfa.17933.1.S1_s_at	DLA-79 (MHC I)	MHC class Ib	**_10.49*_**	<0.001	**_36.99*_**	<0.001	**_9.17*_**	0.004
Cfa.280.1.S1_at	DLA-88 (MHC I)	MHC class I DLA-88	**_17.87*_**	0.018	**_135.55*_**	<0.001	-4.80	0.336
Cfa.18297.1.S1_at	DLA-DMA (MHC II)	major histocompatibility complex, class II, DM alpha	_3.86*_	<0.001	**_6.35*_**	<0.001	**_4.48*_**	0.009
Cfa.21014.2.S1_s_at	DLA-DMB (MHC II)	major histocompatibility complex, class II, DM beta	**_4.20*_**	<0.001	**_5.88*_**	0.007	**_4.88*_**	0.018
CfaAffx.2154.1.S1_at	DLA-DOB (MHC II)	major histocompatibility complex, class II, DO beta	_1.41*_	0.013	_2.46*_	<0.001	1.07	0.055
Cfa.182.1.S2_at	DLA-DQA1 (MHC II)	major histocompatibility complex, class II, DQ alpha 1	**_12.19*_**	<0.001	**_30.97*_**	<0.001	**_17.87*_**	0.004
CfaAffx.2152.1.S1_s_at	DLA-DQB1 (MHC II)	major histocompatibility complex, class II, DQ beta 1 /// HLA class II histocompatibility antigen, DQ beta 2 chain-like	**_13.34*_**	<0.001	**_22.98*_**	<0.001	**_14.09*_**	0.004
Cfa.6456.1.S1_at	DLA-DRA (MHC II)	MHC class II DR alpha chain	**_5.27*_**	<0.001	**_8.34*_**	0.003	**_6.78*_**	0.009
Cfa.181.1.S1_at	DLA-DRB1 (MHC II)	MHC class II DLA DRB1 beta chain	**_6.65*_**	<0.001	**_9.77*_**	0.001	**_7.64*_**	0.004

Shown are fold changes in acute, subacute and chronic lesions including *P* values based on Mann-Whitney U tests. Significant fold changes are shown in italics letters and marked with an asterisk. Bold letters indicate fold changes higher than 4.0. °Canine IFNA5 is identical to canine IFNA3, IFNA4, IFNA6, and IFNA8 as described in Klotz et al. (2017).

Klotz, D.; Baumgärtner, W.; Gerhauser, I., Type I interferons in the pathogenesis and treatment of canine diseases. *Vet. Immunol. Immunopathol.* **2017,** 191, 80-93.

Table S2. Anamnestic data of the investigated dogs.

Animal Number	Breed	Infection Status	Age	Gender
1	Bobtail	CDV	4 months	female
2	Mix	CDV	5 months	female
3	West Highland Terrier	CDV	3 months	unknown
4	Maltese	CDV	5 months	male
5	Siberian Husky	CDV	12 months	male
6	Miniature Pinscher	CDV	7 years	male
7	Mix	CDV	46 months	female
8	Podenco	CDV	juvenile	female
9	Mix	CDV	12 months	female
10	English Bulldog	CDV	3 months	female

11	Mix	CDV	2 months	male
12	Mix	CDV	unknown	female
13	Mix	CDV	unknown	male
14	Mix	CDV	unknown	male
15	Chihuahua	CDV	36 months	female
16	Bernese Mountain dog	Control	15 months	male
17	French Bulldog	Control	2 months	male
18	Rottweiler	Control	6 months	female
19	Beagle	Control	20 months	male
20	Beagle	Control	17 months	female
21	Beagle	Control	unknown	unknown
